# Association of COVID 19 pandemic with new onset Obsessive-Compulsive Disorder (OCD) symptomology in the medical students – A cross sectional study

**DOI:** 10.1192/bjo.2021.718

**Published:** 2021-06-18

**Authors:** Khadija Mazhar, Fatima Khaliq, Daneyal Arshad

**Affiliations:** Rawalpindi Medical University

## Abstract

**Aims:**

Obsessive-Compulsive Disorder (OCD) is an anxiety disorder, which is the sixth largest contributor to non-fatal health loss globally. Coronavirus disease (COVID-19) pandemic, aside from its impact on physical health, has also had its effects on mental health. This study aimed to explore the frequency of new onset OCD symptomology in medical students amidst COVID-19 pandemic and its association with potential sociodemographic parameters.

**Method:**

This cross-sectional study was conducted among medical students studying in Pakistani medical colleges. Data were collected after ethical approval from 1st January 2020 to 20th January 2020 during the second COVID-19 wave. Participants with a history of diagnosed psychiatric illness such as OCD, bipolar disorder, depression, anxiety and those taking relevant medications were excluded from the study. The online questionnaire included Yale-Brown Obsessive-Compulsive Scale (Y-BOCS) and Revised Padua Inventory-Contamination Subscale (PI-CS), which were used to assess OCD symptoms and aversion for contamination respectively. Participants filled Y-BOCS twice, once for pre-pandemic score (based on self-recall), and a second time during 2nd wave of COVID-19. Data were analysed using IBM SPSS v23.0 (Armonk, N.Y., USA).

**Result:**

The study included 711 participants (Males: 29.8%, Mean age: 21.59 ± 1.52 years) from over 46 medical colleges and over 44 cities of Pakistan. The mean pre-pandemic and mid-2nd wave Y-BOCS scores were 11.86 ± 6.02 and 15.61 ± 7.41 respectively. The mean PI-CS score was 17.27 ± 9.17. Twenty five percent (n = 176) of students developed new onset OCD symptomology during pandemic, while seventy percent (n = 497) suffered from worsened Y-BOCS score during pandemic. New onset OCD symptomology was associated with age less than 20 years (p = 0.02), higher PI-CS score (p = 0.001) and studying in preclinical years (p = 0.002). Worsening of YBOCS score had significant association with female gender (p = 0.02), attending pandemic related awareness seminar (p = 0.027), studying in preclinical years (p < 0.001) and age less than 20 years (p < 0.001). High Padua scorers (16 and above) showed significant association with increase in YBOCS score (p < 0.000), age less than 20 years (p = 0.005), preclinical years (p = 0.001), frequency of engagements in pandemic related discussions (p = 0.001) and change in YBOCS score (p < 0.001).

**Conclusion:**

Our findings indicate that the prevalence of OCD symptomology increased during the COVID-19 pandemic as demonstrated by increased Y-BOCS scores.Femal medical students and students in preclinical years are more likely to suffer from psycological impact of COVID-19 pandemic and heightened concerns and fear for contamination.

